# Aging, the microbiota–gut–brain axis, and late-life epilepsy: a hypothesis-driven review

**DOI:** 10.3389/fnmol.2026.1861519

**Published:** 2026-09-14

**Authors:** Yingsi Cao, Huiying Li, Minzheng Xu, Yuting Teng, Hai-Ying Gu

**Affiliations:** School of Health Medicine, Nantong Institute of Technology, Nantong, Jiangsu, China

**Keywords:** aging, gut dysbiosis, late-life epilepsy, microbiota–gut–brain axis, neuroinflammation

## Abstract

Late-life epilepsy is an increasingly important neurological and public health challenge, yet its biological basis remains incompletely understood. The microbiota–gut–brain axis has emerged as a systems-level framework linking peripheral metabolism, barrier integrity, immune signaling, and brain excitability. Growing evidence supports an association between gut dysbiosis and epilepsy, particularly drug-resistant epilepsy, although direct evidence specifically addressing this axis in late-life epilepsy remains limited. Most available data instead come from aging biology, general epilepsy cohorts, pediatric populations, and preclinical models. This review considers how aging may reshape the microbiota–gut–brain axis in ways relevant to epilepsy in older adults. We summarize aging-related remodeling of this axis, including gut dysbiosis, impaired intestinal and blood–brain barrier/neurovascular unit homeostasis, loss of protective microbial metabolites, chronic low-grade inflammation, and neuroimmune priming. We then review clinical, functional, and mechanistic evidence linking microbiota-related abnormalities to epilepsy, with emphasis on broad ecological imbalance, barrier dysfunction, neuroinflammatory signaling, short-chain fatty acid pathways, and vagal gut–brain communication. On this basis, we propose that aging may increase the likelihood that epilepsy-associated microbiota–gut–brain axis abnormalities translate into persistent peripheral inflammation, BBB/NVU vulnerability, amplified neuroinflammation, and reduced neural network resilience, thereby increasing seizure susceptibility. We further discuss microbiota-targeted interventions, including ketogenic diet, probiotics and prebiotics, fecal microbiota transplantation, and metabolite-based strategies, as hypothesis-informed translational directions rather than established therapies for late-life epilepsy. Overall, we suggest that the microbiota–gut–brain axis functions as a context-dependent modifier of vulnerability in late-life epilepsy and provides a useful framework for guiding future age-stratified, biomarker-oriented, and etiology-aware studies.

## 1 Introduction

Late-life epilepsy generally refers to epilepsy with onset at or after 60 years of age, although some epidemiological and geriatric studies use 65 years as an alternative cutoff because it corresponds to conventional older-adult cohort definitions (Beghi and Giussani, 2018). As populations age worldwide, late-life epilepsy has become an increasingly important neurological and public health challenge (Wang Z. et al., 2025; Dong et al., 2026; Zawar et al., 2026). Older adults with epilepsy frequently present with multimorbidity, polypharmacy, cerebrovascular disease, increased dementia risk, and functional vulnerability, indicating that epilepsy in later life is biologically and clinically distinct from epilepsy earlier in life (Eberhart et al., 2025; Helmstaedter et al., 2025; Thacker et al., 2025). These features support broader mechanistic frameworks extending beyond neuron-centered explanations alone.

The microbiota–gut–brain axis has emerged as a systems-level interface linking microbial metabolism, immune signaling, barrier homeostasis, and neural excitability. Current reviews and meta-analyses support an association between gut dysbiosis and epilepsy, particularly drug-resistant epilepsy (DRE), defined by the International League Against Epilepsy as failure of adequate trials of two tolerated, appropriately chosen, and properly used antiseizure medication schedules to achieve sustained seizure freedom (Kwan et al., 2010). Experimental and translational studies further suggest that microbiota-related signals may influence seizure susceptibility through interconnected inflammatory, metabolic, neuroimmune, and barrier-related mechanisms (Federici et al., 2025; Karakis, 2025; Kowalcze et al., 2025; Ravizza et al., 2025; Tan et al., 2025; Yan et al., 2025).

Aging may be particularly relevant to this axis because it alters both peripheral and central mechanisms involved in microbial, immune, and neurovascular homeostasis long before seizure onset. Peripherally, aging is associated with reduced microbial resilience, altered metabolite production, impaired gut barrier integrity, and chronic low-grade inflammation (Escudero-Bautista et al., 2024; Le Cosquer et al., 2024; Pan et al., 2025; Kim, 2026). Within the central nervous system, aging is linked to reduced neurovascular resilience, microglial priming, neurovascular unit (NVU) dysfunction, and heightened inflammatory responsiveness (Knox et al., 2022; Andjelkovic et al., 2023; Kim et al., 2025; Santisteban and Iadecola, 2025; Glavan et al., 2026). These alterations may increase the likelihood that peripheral inflammatory and metabolic disturbances influence seizure-related neural networks in older adults.

Direct evidence specifically addressing the microbiota–gut–brain axis in late-life epilepsy remains limited. Most available data derive from aging biology, general epilepsy cohorts, pediatric populations, and preclinical models rather than dedicated cohorts of older adults with epilepsy (Federici et al., 2025; Karakis, 2025; Kowalcze et al., 2025; Tan et al., 2025; Yan et al., 2025). This review therefore focuses on how aging may amplify microbiota–gut–brain axis abnormalities relevant to epilepsy, with particular emphasis on dysbiosis, barrier dysfunction, neuroinflammation, neurovascular vulnerability, and microbiota-targeted interventions. Throughout the review, direct evidence is distinguished from hypothesis-generating mechanistic inference, and the principal claims are summarized in Table 1.

**Table 1:** Evidence strength of the main claims discussed in this review.

Key claim	Main evidence base	Evidence strength	Main limitation
Limited direct evidence in late-life epilepsy	Aging biology, general epilepsy cohorts, pediatric studies, and preclinical models	Limited direct evidence	Dedicated cohorts of older adults with epilepsy are lacking
Broad dysbiosis in epilepsy	Clinical studies and meta-analyses in epilepsy cohorts	Moderate human evidence	Marked inter-cohort heterogeneity
Functional microbiota effects on seizure phenotypes	Animal intervention studies, antibiotics, probiotics, and FMT models	Moderate preclinical evidence	Most evidence comes from experimental models
Aging-related dysbiosis, barrier vulnerability, and inflammaging	Aging biology, human microbiome studies, and preclinical aging models	Moderate aging-related evidence	Evidence is not epilepsy-specific
Aging-amplified consequences of epilepsy-associated dysbiosis	Integrated evidence from aging biology, epilepsy research, and mechanistic studies	Hypothesis-generating indirect evidence	No direct validation in dedicated late-life epilepsy cohorts
Microbiota-targeted interventions in late-life epilepsy	Ketogenic diet, probiotics/prebiotics, FMT, and metabolite-based interventions	Limited translational evidence	Dedicated late-life epilepsy trials are lacking

FMT, fecal microbiota transplantation.

## 2 Aging-related remodeling of the microbiota–gut–brain axis

Aging is associated with microbial remodeling, impaired barrier homeostasis, and chronic low-grade inflammation across the microbiota–gut–brain axis. Most evidence currently derives from aging biology, population-based microbiome studies, and preclinical models rather than dedicated cohorts of late-life epilepsy. These alterations may increase susceptibility to epilepsy-related inflammatory and metabolic disturbances in older adults (Thevaranjan et al., 2017; Wilmanski et al., 2021; López-Otín et al., 2023; Dingledine et al., 2024; Cummins et al., 2025).

### 2.1 Aging-related gut dysbiosis

Aging-related dysbiosis is increasingly linked to altered microbial composition, reduced ecological resilience, and impaired metabolic output in older adults (Ma et al., 2024b; Ravikrishnan et al., 2024). Multi-cohort studies suggest that aging-related microbial remodeling is more consistently reflected by altered metabolic function and reduced ecological resilience than by uniform taxonomic shifts across populations (Ravikrishnan et al., 2024; Xia et al., 2024).

Importantly, aging is not uniformly associated with dysbiosis. Some healthy older adults retain microbial diversity or develop increased microbial uniqueness associated with favorable metabolic profiles, whereas unhealthy aging is more often linked to depletion of commensals and enrichment of inflammation-associated taxa (Wilmanski et al., 2021; Ghosh et al., 2022; Fu et al., 2023). Aging-related dysbiosis should therefore be viewed as a risk-associated trajectory rather than an inevitable consequence of chronological aging.

One of the most reproducible aging-related patterns is depletion of health-associated commensals and SCFA-producing taxa, including *Bifidobacterium*, *Lactobacillus*, and multiple butyrate-producing bacteria (Odamaki et al., 2016; Chen et al., 2024; Xia et al., 2024). Among short-chain fatty acids (SCFAs), butyrate currently has the strongest evidence for maintaining intestinal and BBB integrity and for modulating inflammation-related pathways relevant to epilepsy, whereas epilepsy-specific evidence for propionate and valerate remains more limited (Lee et al., 2020; Wang et al., 2021; Fock and Parnova, 2023). Loss of SCFA-producing bacteria may therefore contribute to impaired barrier support and chronic low-grade inflammation in aging (Odamaki et al., 2016; Lee et al., 2020; Wang et al., 2021).

Aging-related dysbiosis is also associated with altered bile acid metabolism and impaired carbohydrate utilization pathways, favoring a more inflammation-prone intestinal environment (Sato et al., 2021; Chen et al., 2024; Ravikrishnan et al., 2024). Compared with SCFA-related mechanisms, however, the role of bile acid signaling in epilepsy remains less directly established and is therefore considered here as a secondary metabolic pathway rather than a central mechanistic axis.

### 2.2 Leaky gut and inflammaging

Aging-related dysbiosis is frequently accompanied by increased intestinal permeability, chronic low-grade inflammation, and persistent exposure to microbiota-associated inflammatory signals (Franceschi et al., 2018; López-Otín et al., 2023). Aging-related disruption of tight-junction homeostasis, mucus-layer integrity, and epithelial barrier function may facilitate translocation of microbial products such as lipopolysaccharide (LPS) into the circulation (Salazar et al., 2023; Dmytriv et al., 2024; Neurath et al., 2025), thereby sustaining systemic inflammatory activation through TLR4/MyD88/TRIF–NF-κB signaling pathways (Kim et al., 2023; Yan et al., 2024). Altered localization or reduced expression of tight-junction proteins including occludin, ZO-1, and claudins may further contribute to aging-related barrier vulnerability (Horowitz et al., 2023; Wang and Yang, 2024). In this context, dysbiosis, leaky gut, and inflammaging are better viewed as interconnected processes rather than independent phenomena.

Because direct measurement of circulating LPS is methodologically challenging, LPS-binding protein (LBP) is commonly used as a surrogate marker of persistent low-grade endotoxin exposure (Sun et al., 2010). Aging may further amplify these responses because aged mice exhibit increased inflammatory and endothelial activation following low-dose LPS exposure, whereas microbiota derived from aged donors has been associated with heightened endotoxemia-associated inflammatory phenotypes and increased TLR4-related signaling in recipient animals (Caetano-Silva et al., 2024; Suraj-Prażmowska et al., 2025). Together, these findings suggest that aging may increase host responsiveness to microbiota-associated inflammatory signaling rather than merely increasing basal inflammation.

### 2.3 BBB/NVU vulnerability and loss of protective metabolites

Peripheral inflammatory signaling may influence central immune responses through blood–brain barrier (BBB) and neurovascular unit (NVU) dysfunction together with glial activation (Greene et al., 2024; Beltran-Velasco and Clemente-Suárez, 2025). In aging, BBB and NVU dysfunction more commonly manifest as reduced barrier resilience and increased sensitivity to inflammatory and metabolic stress rather than persistent baseline leakage (Cummins et al., 2025). Neuroinflammaging is associated with microglial priming, astrocytic reactivity, BBB/NVU dysfunction, and increased participation of peripheral immune signaling (Kaur et al., 2025). Together, these changes may increase the susceptibility of the aged brain to inflammatory activation.

Peripheral inflammatory mediators may further promote endothelial activation, immune-cell recruitment, and glial inflammatory responses even in the absence of overt BBB breakdown (Wang et al., 2015; Paré et al., 2018; Mai et al., 2021; Burkhart et al., 2024). These findings suggest that aging-related peripheral inflammation may more readily influence central immune signaling under conditions of reduced neurovascular resilience. Consistent with this view, peripheral immune remodeling can influence neuroinflammatory responses even without overt BBB breakdown (Schroer et al., 2023).

Microbiota-derived metabolites also contribute to BBB stability. Germ-free animal studies demonstrated increased BBB permeability, whereas recolonization partially restored barrier integrity together with improved expression of tight-junction proteins including occludin, claudin-5, and ZO-1 (Braniste et al., 2014). Among microbiota-derived metabolites, short-chain fatty acids (SCFAs) have been linked to maintenance of BBB homeostasis through stabilization of tight junctions and suppression of inflammatory signaling (Fock and Parnova, 2023; Veerareddy et al., 2025).

Together, the findings discussed across Section 2 suggest that aging-related dysbiosis, barrier dysfunction, neurovascular vulnerability, and loss of protective microbial metabolites may collectively increase susceptibility to neuroinflammatory and seizure-related network disturbances in older adults (Figure 1).

**Figure 1:**
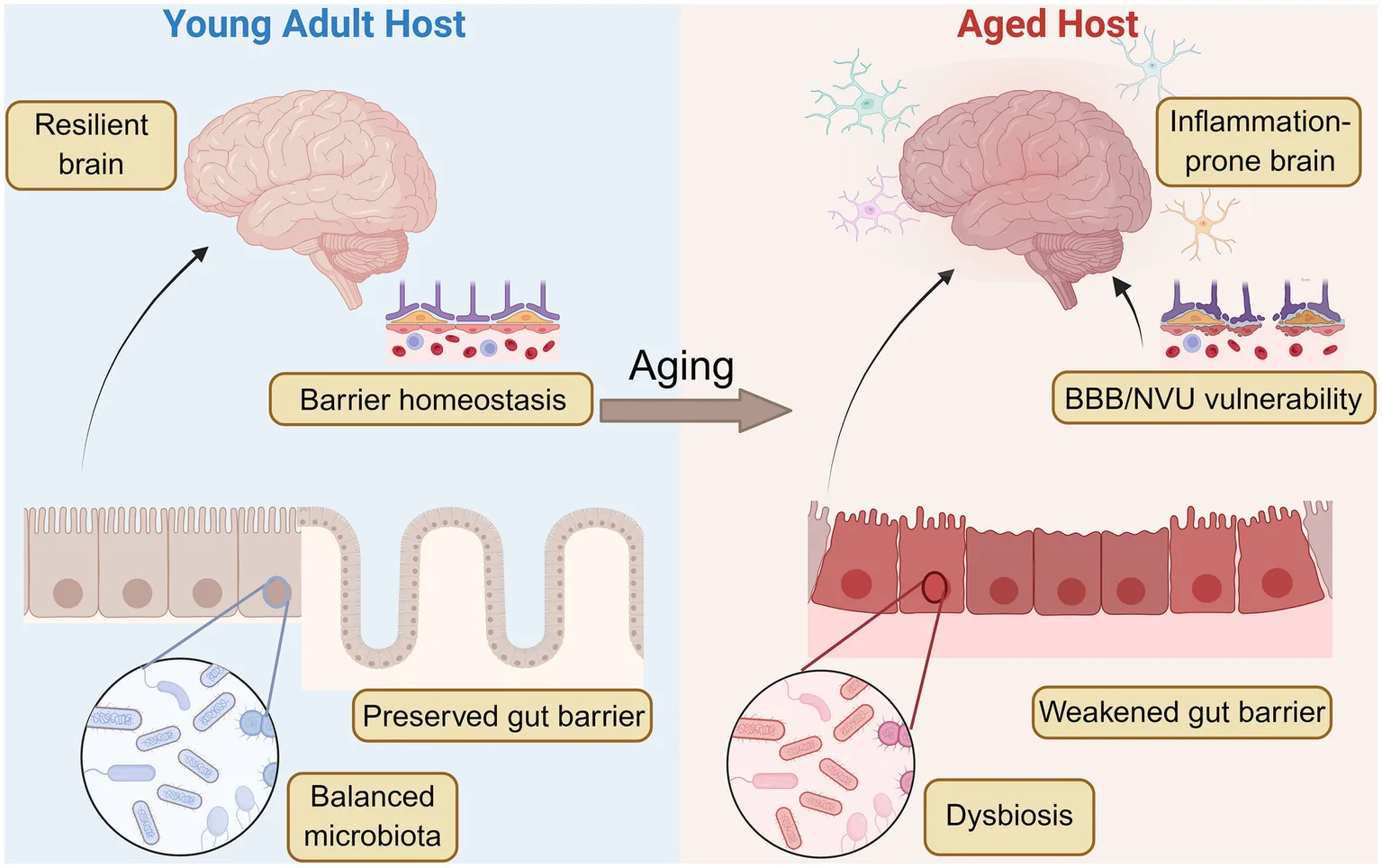
Aging-related remodeling of the microbiota–gut–brain axis. Aging is associated with progressive changes in gut microbial composition, reduced microbial resilience, impaired intestinal barrier integrity, altered microbial metabolite profiles, and increased neuroimmune vulnerability. These components are supported by evidence from human aging studies and experimental models. Their potential contribution to epilepsy susceptibility represents a hypothesis-driven framework requiring further validation.

## 3 Microbiota–gut–brain axis abnormalities in epilepsy

The aging-related alterations outlined above raise a focused question: whether microbiota–gut–brain axis abnormalities observed in epilepsy may exert greater biological consequences in older adults. Current evidence suggests that epilepsy is associated not only with abnormal neuronal excitability, but also with alterations in gut microbial ecology, microbial metabolites, barrier-related pathways, and inflammatory signaling. Clinical studies mainly support association, whereas experimental studies provide stronger functional and mechanistic evidence for microbiota involvement in seizure-related phenotypes (Federici et al., 2025; Karakis, 2025; Kowalcze et al., 2025; Ravizza et al., 2025; Tan et al., 2025; Yan et al., 2025). Dedicated microbiota cohorts in late-life epilepsy remain limited. This section therefore summarizes epilepsy-associated microbiota–gut–brain axis abnormalities as a mechanistic foundation for the aging-related framework discussed in later sections.

### 3.1 Clinical evidence

Clinical studies and meta-analyses generally support the presence of gut microbial dysbiosis in epilepsy, although the evidence more consistently indicates broad ecological and functional imbalance than a single reproducible microbial signature (Yang R. et al., 2024; Federici et al., 2025). Reported alterations in α-diversity, β-diversity, and taxonomic composition vary across cohorts, likely reflecting differences in epilepsy subtype, etiology, diet, age structure, and antiseizure medication exposure (Fang et al., 2025; Ottaviano et al., 2025; Riva et al., 2025). Epilepsy-associated dysbiosis is therefore better viewed as a heterogeneous disturbance of microbial ecology and metabolic function rather than a fixed taxonomic pattern.

Despite this heterogeneity, several directional trends have emerged. Many cohorts report reduced abundance of multiple commensal or SCFA-producing taxa, including Ruminococcaceae, Faecalibacterium, Roseburia, and Blautia, together with relative enrichment of taxa associated with inflammatory or dysbiotic states, such as Proteobacteria and Verrucomicrobia in some populations (Yang R. et al., 2024; Mousavi et al., 2025; Ottaviano et al., 2025; Riva et al., 2025). These alterations are likely more relevant as indicators of impaired metabolic and inflammatory homeostasis than as disease-specific microbial markers. Collectively, they suggest reduced protective metabolite production, impaired barrier support, and increased inflammatory signaling in epilepsy-associated microbial communities.

Microbiota-related changes may also track disease complexity. Drug resistance, comorbidity burden, cognitive impairment, and treatment-response variability have each been associated with more pronounced microbial remodeling in some studies (Zhou et al., 2022; Hong, 2024; Shariatmadari et al., 2024; Fang et al., 2025; Wang et al., 2026). Although these observations do not establish causality, they support the possibility that microbiota-related abnormalities may contribute to clinical heterogeneity in epilepsy rather than merely accompany seizure diagnosis.

### 3.2 Functional evidence

Compared with clinical association studies, experimental interventions, including fecal microbiota transplantation (FMT) and microbiota depletion approaches, provide stronger functional support for microbiota involvement in epilepsy-related phenotypes. These studies suggest that microbiota-related processes can influence seizure severity together with inflammatory, metabolic, and barrier-related pathways rather than merely accompany seizures.

Multiple experimental models support this interpretation. In acute pentylenetetrazole (PTZ) and chronic epilepsy models, microbiota-targeted interventions, including probiotic treatment, trioctanoin, and butyrate-producing bacterial supplementation, have been associated with reduced seizure burden together with improvements in inflammatory, oxidative stress, and barrier-related measures (Aygun et al., 2022; Ciltas et al., 2023; Yu et al., 2025; Li et al., 2026). Antibiotic depletion studies provide complementary evidence because disruption of the gut microbiota weakens or abolishes some protective intervention effects, suggesting partial dependence on an intact microbial ecosystem (Erickson et al., 2025). FMT studies provide more direct evidence for functional transferability. In the WAG/Rij absence epilepsy model, microbiota derived from epileptic donors influenced seizure-related phenotypes in recipient animals, supporting the view that at least some epilepsy-associated microbial features are functionally transferable (Citraro et al., 2021).

Current functional evidence therefore supports the biological relevance and experimental modifiability of microbiota-related pathways in epilepsy. However, the mechanisms underlying these effects and their relevance to late-life epilepsy remain incompletely defined (Caetano-Silva et al., 2024; Cummins et al., 2025).

### 3.3 Mechanistic interfaces

Current mechanistic evidence supports several interconnected pathways through which microbiota-related disturbances may influence seizure susceptibility, including neuroactive metabolite signaling, SCFA-related barrier regulation, immune-inflammatory activation, and vagus-associated gut–brain communication. Although these mechanisms have not been specifically validated in late-life epilepsy, they identify biological interfaces likely to interact with aging-related neuroimmune and neurovascular vulnerability.

#### 3.3.1 Neuroactive metabolites and excitatory–inhibitory balance

Certain *Lactobacillus* and *Bifidobacterium* species possess GABA-producing capacity through the gadB/gadC pathway (Barrett et al., 2012; Yunes et al., 2016). However, current evidence does not support a simple direct effect of bacterially derived GABA on seizure threshold. A more plausible interpretation is that microbiota-related metabolites indirectly influence seizure-related network excitability through immune, endocrine, metabolic, or vagal pathways (Bravo et al., 2011; Yunes et al., 2020; Loh et al., 2024; Belelli et al., 2025). These interactions may become more relevant in aging because the aged brain exhibits greater inflammatory and neurovascular responsiveness.

#### 3.3.2 The SCFA–barrier–neuroinflammation axis

Among current mechanistic pathways, SCFA-related signaling, particularly butyrate-related pathways, represents one of the most plausible interfaces linking microbial alterations, barrier dysfunction, neuroinflammation, and seizure susceptibility.

Although multiple SCFAs contribute to host metabolic and immune regulation, butyrate receives the greatest emphasis in this review because it currently has the strongest evidence for maintaining intestinal and blood–brain barrier integrity, suppressing inflammatory signaling, and modulating epilepsy-related phenotypes. By contrast, epilepsy-specific evidence for valerate remains limited (Braniste et al., 2014; Lee et al., 2020; Majumdar et al., 2024; Zhai et al., 2024).

Epilepsy-associated dysbiosis is frequently accompanied by depletion of SCFA-producing taxa and impaired protective metabolic signaling (Smith et al., 2013; Yan et al., 2025). Experimental studies further suggest that butyrate-related interventions can attenuate neuroinflammatory signaling and delay epileptogenesis in seizure models (Majumdar et al., 2024; Zhai et al., 2024). Additional microbiota-targeted studies further support links among microbial metabolites, barrier integrity, inflammatory signaling, and seizure-related phenotypes (Li et al., 2026).

#### 3.3.3 Vagal signaling and gut–brain circuits

Vagus-associated gut–brain signaling provides a non-blood-borne route through which microbiota-related signals may influence brain function. *Lactobacillus rhamnosus* alters brain GABA receptor expression through a vagus-dependent mechanism (Bravo et al., 2011). Direct epilepsy-specific evidence remains limited, but the relevance of this pathway is supported by the established anti-seizure effects of vagus nerve stimulation (Fülling et al., 2019; Faraji et al., 2025; Federici et al., 2025; Jameson et al., 2025). Collectively, these findings support the possibility that vagus-associated signaling contributes to communication between microbiota-related signals and seizure-related neural circuits.

### 3.4 Bidirectional interactions

Recurrent seizures may themselves influence the microbiota–gut–brain axis through stress signaling, autonomic dysfunction, altered intestinal motility, systemic inflammation, and barrier disruption (Medel-Matus et al., 2018; Lum et al., 2020; Ding et al., 2021; Mengoli et al., 2023; Yan et al., 2025). Microbiota abnormalities in epilepsy are therefore unlikely to represent a purely upstream phenomenon and may instead participate in bidirectional pathological interactions.

Experimental studies support this interpretation. Acute and chronic seizure models have been associated with increased intestinal permeability, inflammatory activation, endotoxemia-related signaling, and alterations in gut microbial composition (Medel-Matus et al., 2018; De Caro et al., 2019; Zou et al., 2026). These findings suggest that recurrent seizures may secondarily remodel the intestinal environment and reinforce inflammatory and barrier-related disturbances associated with epilepsy-related dysbiosis. Nevertheless, the relative contribution of microbiota-driven versus seizure-driven alterations remains incompletely defined.

Antiseizure medications (ASMs) may further complicate this relationship. Several ASMs have been associated with altered microbial composition together with gastrointestinal and metabolic disturbances (Ilhan et al., 2022; Thai et al., 2023; Dop et al., 2024). This interaction may be particularly relevant in older adults, in whom age-related changes in hepatic metabolism, renal clearance, and drug disposition, together with polypharmacy, may alter ASM exposure and potentially modify ASM-associated microbial disturbances (McCarthy et al., 2026). Conversely, gut microbial alterations may influence drug disposition through microbial biotransformation and modulation of intestinal drug metabolism or absorption (Wang X. et al., 2025). Whether these interactions meaningfully affect ASM pharmacokinetics in older adults remains unknown, but they could contribute to interindividual variability in ASM exposure and treatment response, representing a potential pharmacomicrobiomic mechanism relevant to drug resistance. Consequently, microbiota alterations observed in older adults with epilepsy may reflect the combined effects of seizures, medications, aging, and comorbid disease rather than epilepsy alone.

Together, current evidence suggests that microbiota-related pathways in epilepsy are biologically relevant, experimentally modifiable, and unlikely to operate through purely unidirectional mechanisms. Although available data remain insufficient to establish an age-specific microbial model of late-life epilepsy, aging-related neuroimmune and barrier vulnerability may amplify the neurological consequences of epilepsy-associated dysbiosis. A schematic summary of the principal mechanisms discussed in this section is shown in Figure 2.

**Figure 2:**
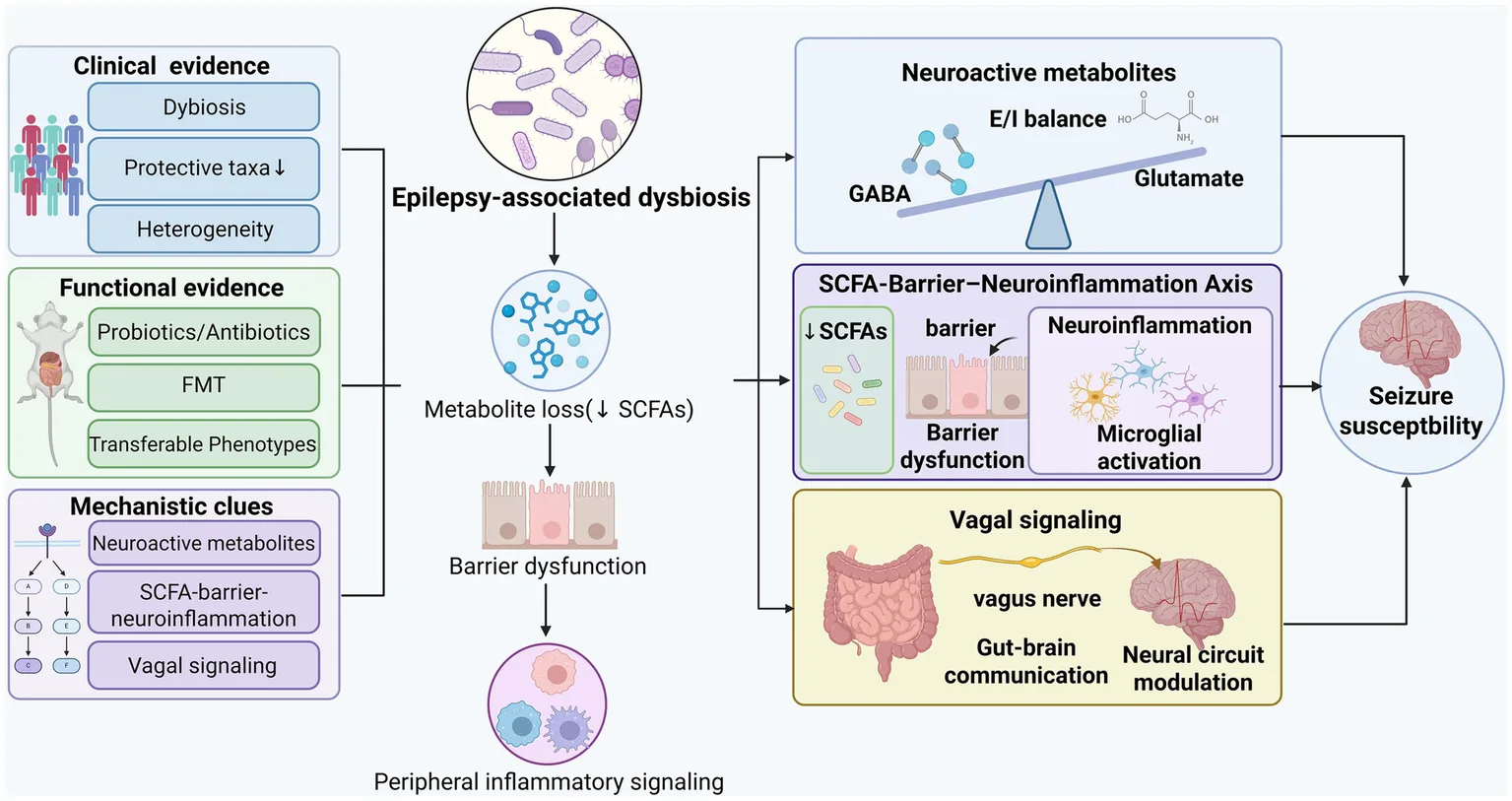
Microbiota–gut–brain axis abnormalities in epilepsy. Clinical studies support associations between epilepsy and gut microbial dysbiosis, whereas experimental studies provide functional evidence linking microbiota-related alterations with seizure phenotypes, barrier dysfunction, inflammatory signaling, and metabolic changes. Mechanistic pathways involving neuroactive metabolites, SCFA-related signaling, and vagal communication are supported by varying levels of evidence, while their specific contribution to late-life epilepsy remains to be established.

## 4 Aging amplifies microbiota–gut–brain axis dysregulation in epilepsy

Dedicated studies of the microbiota–gut–brain axis in late-life epilepsy remain limited. This section therefore does not propose an established age-specific causal model. Instead, it examines how aging may modify the biological consequences of epilepsy-associated microbial abnormalities by integrating evidence from aging biology, epilepsy research, and preclinical models.

### 4.1 Aging-related dysbiosis as a vulnerable host background

Aging-related dysbiosis may create a biological context in which microbial, barrier, and inflammatory homeostasis becomes more difficult to maintain. Although aging-related and epilepsy-associated microbial alterations are not identical at the taxonomic level, both are frequently linked to reduced SCFA-producing bacteria, impaired barrier support, and increased inflammatory signaling (Chen et al., 2024; Ravikrishnan et al., 2024; Yang R. et al., 2024; Mousavi et al., 2025). In older adults, these overlapping functional disturbances may be less effectively compensated for because microbial resilience, metabolic buffering, and barrier stability are already reduced. Table 2 summarizes the major overlapping and divergent microbiota-related features associated with aging and epilepsy.

**Table 2:** Comparison of gut microbiota alterations associated with aging and epilepsy and their potential convergence in late-life epilepsy.

Microbiota-related feature	Healthy elderly/aging-associated trends	General adult/pediatric epilepsy	Potential interaction in late-life epilepsy
SCFA-producing taxa	Reduced in less healthy aging; may be preserved in healthy aging	Frequently reduced	Convergent reduction; potentially additive
Butyrate-associated taxa	Reduced in some aging-associated dysbiotic states	Faecalibacterium, Roseburia, and other butyrate-associated taxa reduced in several cohorts	Convergent loss of protective metabolic capacity
*Bifidobacterium*/*Lactobacillus*	Frequently reduced in aging-associated dysbiosis	Less consistently reported	Predominantly aging-related; interaction uncertain
Inflammation-associated taxa	Enriched in less healthy aging	Proteobacteria and other dysbiosis-associated taxa enriched in some cohorts	Potentially convergent inflammatory shift
Microbial diversity/resilience	Diversity may be preserved in healthy aging, whereas resilience may decline with less healthy aging	Heterogeneous across cohorts	Reduced aging-related resilience may increase susceptibility to additional perturbations
SCFA-related metabolic function	Reduced in some aging phenotypes	Impaired in epilepsy-associated dysbiosis	Convergent functional deficit; potential amplification

Potential interactions represent hypothesis-driven interpretations based on overlapping aging- and epilepsy-associated patterns rather than direct evidence from late-life epilepsy cohorts.

Their relationship is therefore more likely to involve convergent functional disturbances rather than recurrence of a fixed microbial signature. Current evidence suggests greater convergence in functional deficits than in individual taxonomic changes, with some alterations potentially acting additively or synergistically, whereas clear antagonistic patterns have not been established. Aging itself may place the microbiota–barrier–inflammation axis in a less resilient state, while epilepsy-related factors such as recurrent seizures, sustained inflammatory burden, and long-term antiseizure medication exposure could further contribute to disruptions in microbial composition and metabolic output (Zhu et al., 2024; Khedpande and Barve, 2025; Takeda et al., 2025).

This convergence is particularly apparent in SCFA-related pathways. Aging-associated loss of butyrate-producing bacteria may reduce microbial metabolic reserve, while epilepsy cohorts have reported depletion of SCFA-producing taxa, including Faecalibacterium and Roseburia, together with impaired protective metabolic signaling (Wang et al., 2021; Yang R. et al., 2024; Li et al., 2026). These overlapping changes may therefore be additive and potentially synergistic at the functional level, particularly when reduced SCFA availability occurs in an aging host already characterized by impaired barrier resilience and chronic low-grade inflammation. Compared with SCFA-related mechanisms, the role of bile acid signaling in epilepsy remains less directly established and is therefore considered here as a secondary aging-related metabolic pathway rather than a central mechanistic axis (Sato et al., 2021; Ma et al., 2024a).

Aging does not necessarily cause epilepsy directly. Rather, it may reduce the capacity of the microbiota–barrier–inflammation axis to recover following recurrent seizures, medication exposure, dietary shifts, or systemic inflammation. In this setting, epilepsy-associated disturbances may be more likely to persist and interact with peripheral and central inflammatory pathways. This aging-related microbial background is also shaped by clinical and environmental factors common in later life. Dietary insufficiency and polypharmacy may influence microbial composition and diversity in older adults (Matsumoto et al., 2025; Nagano et al., 2025), while institutionalization may further modify the gut ecosystem through dietary, clinical, and environmental exposures (Jiménez-Arroyo et al., 2025). Poor oral health and swallowing impairment may also influence the gut microbiome, partly through changes in food choice and dietary intake (Mayama et al., 2026; Yoshimura et al., 2026). These factors may overlap with or modify epilepsy-associated microbial changes, further contributing to the heterogeneity of microbiota profiles in older adults with epilepsy.

### 4.2 Neuroinflammation as a key amplifying interface

Neuroinflammation may represent a key interface linking aging-related dysbiosis to increased seizure susceptibility. In aged hosts, peripheral inflammatory signals may be more readily translated into immune activation within the brain, thereby increasing vulnerability to network instability. The central issue is not simply elevated inflammatory tone itself, but the greater responsiveness of the aged brain to peripheral inflammatory challenge.

Aging-related dysbiosis and barrier dysfunction may sustain peripheral inflammatory signaling, while BBB/NVU vulnerability and glial priming may increase the likelihood that these peripheral signals influence the central nervous system (Caetano-Silva et al., 2024; Cummins et al., 2025; Kaur et al., 2025). Compared with younger hosts, the aged brain appears more reactive to inflammatory and metabolic stress, such that peripheral inflammatory input of similar magnitude may induce stronger central immune activation. Omics and single-cell studies further indicate increased proportions of reactive or primed microglial populations with enhanced expression of inflammation-related genes in the aged brain, particularly in vulnerable regions (Jin et al., 2021; Wang L. et al., 2025). Aging-related dysbiosis and chronic peripheral inflammation may further reinforce this primed state, thereby promoting more sustained glial activation in response to peripheral inflammatory stimuli (Brandt et al., 2023; Tamatta et al., 2025).

Neuroinflammatory pathways may directly influence neuronal excitability and seizure threshold. IL-1β enhances NMDA receptor-related signaling through a Src kinase-dependent mechanism, whereas exogenous IL-1β shortens seizure latency and blockade of this pathway attenuates seizure susceptibility (Balosso et al., 2008; Qin et al., 2022; Zhang et al., 2025). Additional inflammatory pathways, including inflammasome-related signaling, have likewise been implicated in seizure maintenance and neuronal injury across epilepsy models (Qin et al., 2022; Zhang et al., 2025). Recurrent seizures can further activate microglia and astrocytes while disrupting glutamate transport and ionic homeostasis, thereby reinforcing a self-sustaining seizure–inflammation loop.

This mechanism may be particularly relevant to epilepsy because seizure generation is highly sensitive to relatively small disturbances in network stability. Alterations in excitatory–inhibitory balance together with impaired glial, barrier, and synaptic homeostasis can directly influence seizure threshold and network synchronization (Seifert et al., 2010; Heinemann et al., 2012; Vezzani et al., 2019; Dingledine et al., 2024). Activated microglia and astrocytes may further alter cytokine signaling, glutamate uptake, ion homeostasis, and synaptic connectivity, thereby potentially facilitating hypersynchronous neuronal activity and epileptogenesis-related processes (Sanz et al., 2024; Gómez-Oliver et al., 2025; Thergarajan et al., 2025). In this context, aging-related neuroimmune priming may not simply increase general neurological vulnerability, but may preferentially reduce the resilience of neural networks already prone to seizure generation.

### 4.3 Etiology-specific considerations in late-life epilepsy

The microbiota–gut–brain axis is unlikely to contribute uniformly across all forms of late-life epilepsy. Different etiological subgroups may involve distinct patterns of interaction among microbial remodeling, barrier dysfunction, systemic inflammation, and neural network vulnerability. Therefore, late-life epilepsy should be considered a biologically heterogeneous condition when interpreting microbiota–gut–brain axis mechanisms. Current evidence remains indirect, but disease-specific inflammatory and metabolic contexts may modify the consequences of microbiota–gut–brain axis alterations (Dingledine et al., 2024; Ravizza et al., 2025).

In post-stroke epilepsy, microbiota-related alterations may interact with vascular inflammation and neurovascular injury. Stroke studies have reported microbial remodeling, reduced microbial resilience, altered SCFA-related pathways, and increased inflammatory signaling in the context of cerebrovascular injury (Honarpisheh et al., 2022; Jeng et al., 2025; Xie et al., 2025). These changes may contribute to intestinal barrier dysfunction, endotoxin-associated inflammation, and BBB vulnerability, suggesting that the microbiota–gut–brain axis may amplify vascular and neuroimmune disturbances in this context.

In Alzheimer’s disease-related epilepsy and other neurodegeneration-associated epilepsies, microbiota-related mechanisms may interact with chronic neuroimmune vulnerability. Altered microbial metabolites and gut-derived inflammatory signals have been linked to microglial activation and synaptic dysfunction in Alzheimer’s disease (Bello-Corral et al., 2023; Yang J. et al., 2024). These processes may further interact with age-related neuroimmune priming and network instability, although direct microbiome studies in Alzheimer’s disease-related epilepsy remain limited.

In autoimmune epilepsy, microbiota-related mechanisms may involve immune regulation and barrier dysfunction-associated inflammatory pathways. Evidence from immune-mediated neurological disorders suggests that microbial alterations may influence systemic immune activation and inflammatory susceptibility, but direct evidence in autoimmune epilepsy remains insufficient (Cryan et al., 2019).

In brain tumor-related epilepsy, microbiota-related effects may be influenced by tumor-associated inflammation, treatment exposure, and metabolic alterations. Cancer microbiome studies indicate that tumor status and anticancer therapies can modify microbial composition and immune signaling, which may interact with the tumor-associated inflammatory milieu and BBB dysfunction (Helmink et al., 2019; Sepich-Poore et al., 2021).

Together, these considerations suggest that the microbiota–gut–brain axis may function as an etiology-dependent modifier rather than a uniform mechanism across late-life epilepsy. Future studies should incorporate etiological stratification to determine whether distinct microbiota–barrier–immune signatures characterize different subtypes of late-life epilepsy.

### 4.4 Conceptual model and testable hypotheses

Taken together, current evidence suggests that aging-related dysbiosis, impaired SCFA-mediated protection, barrier vulnerability, and heightened neuroimmune reactivity may collectively increase the susceptibility of seizure-related neural networks to peripheral inflammatory and metabolic stress. In this framework, impaired intestinal and BBB/NVU homeostasis together with reduced protective metabolic signaling may facilitate persistent peripheral inflammation and endotoxin-associated signaling. At the same time, age-related glial priming and neurovascular reactivity may increase the likelihood that these peripheral signals disrupt excitatory–inhibitory balance and network stability. Recurrent seizures may further reinforce dysbiosis and inflammatory signaling, supporting a bidirectional rather than purely unidirectional pathway.

One testable possibility is a combined microbiota–barrier–inflammation biomarker profile. Older adults with epilepsy, particularly those with drug-resistant disease, may exhibit a combined microbiota–barrier–inflammation signature characterized by reduced fecal SCFAs, depletion of SCFA-producing taxa, elevated LBP/LPS-related markers, inflammatory cytokines, and BBB-associated injury markers. This possibility could be evaluated in age-matched cross-sectional cohorts comparing late-life epilepsy, drug-responsive epilepsy, drug-resistant epilepsy, and non-epileptic controls.

A second hypothesis is that baseline microbial and metabolic features may contribute to inter-individual variability in treatment response or susceptibility to drug resistance. Relevant features may include fecal SCFA profiles, microbial diversity and resilience indices, LBP, and inflammatory cytokines. This possibility warrants evaluation in prospective longitudinal cohorts sampled before antiseizure medication initiation or treatment adjustment.

A related age-interaction hypothesis is that microbiota-targeted interventions may exert age-dependent effects, with aged hosts showing greater vulnerability to microbiota-related inflammatory and barrier disturbances than younger counterparts. Potential approaches include modulation of butyrate-related pathways, barrier-protective strategies, probiotics, or FMT combined with donor-age manipulation. Gnotobiotic, antibiotic-controlled, and seizure-induction models across young and aged hosts may further help distinguish microbiota-dependent effects from seizure-driven and host-age-dependent alterations.

Given the absence of standardized microbiome cohorts in late-life epilepsy, these hypotheses are currently better interpreted as directional rather than quantitative. Quantitative thresholds or minimum expected effect sizes should therefore be empirically derived rather than prespecified at this stage. Nevertheless, they provide a more focused framework for investigating how aging-related alterations in the microbiota–gut–brain axis may influence seizure susceptibility and disease complexity in older adults. Figure 3 summarizes the proposed aging-amplification model, in which aging-related dysbiosis, barrier vulnerability, and neuroimmune priming may increase the biological consequences of epilepsy-associated microbiota–gut–brain axis disturbances.

**Figure 3:**
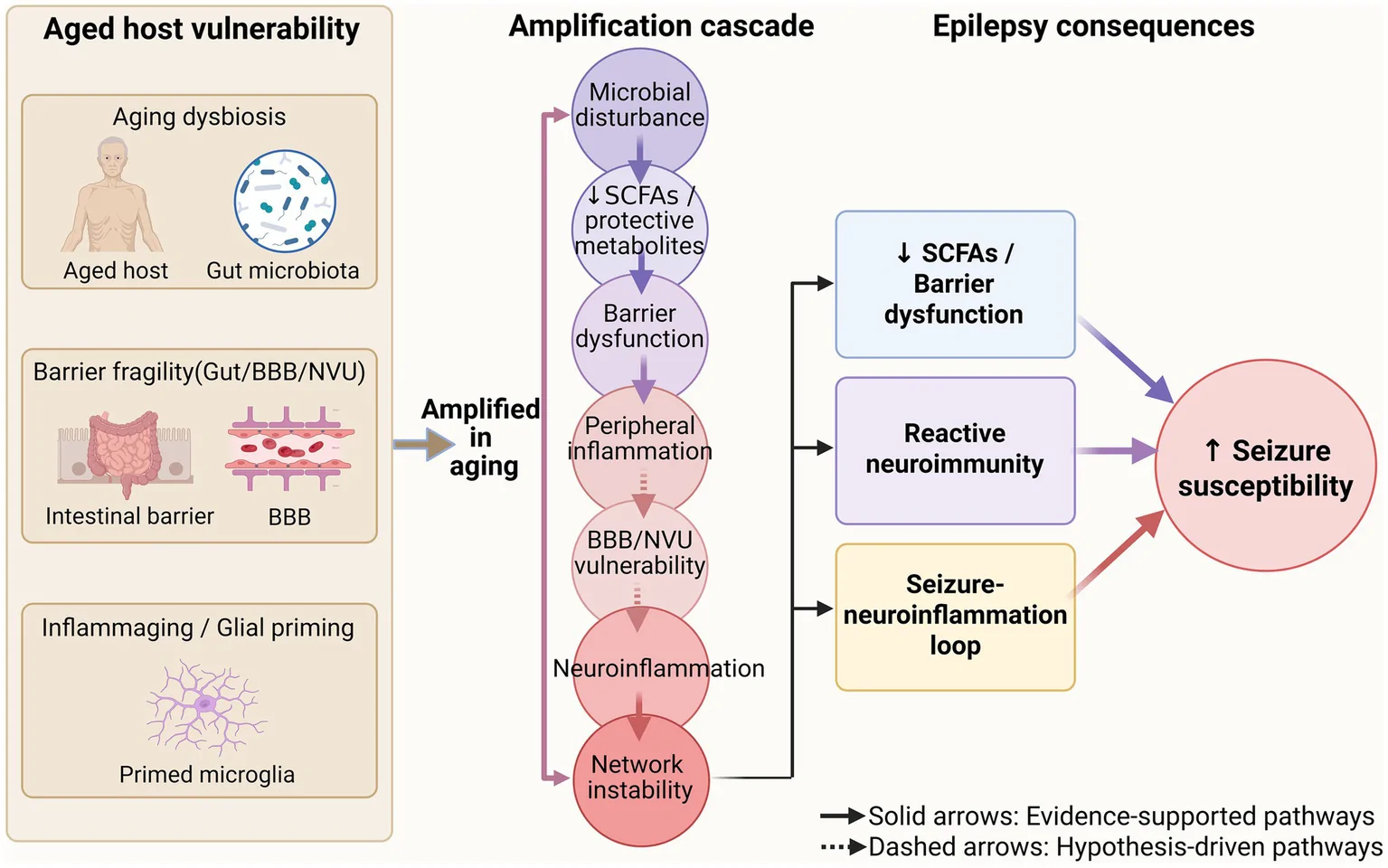
Proposed framework for aging-amplified microbiota–gut–brain axis dysregulation in epilepsy. Aging may increase the impact of epilepsy-associated microbiota disturbances by creating a vulnerable host background characterized by dysbiosis, barrier fragility (gut barrier and BBB/NVU), and glial priming. These changes may amplify a cascade involving barrier dysfunction, peripheral inflammation, BBB/NVU vulnerability, neuroinflammation, and network instability, ultimately increasing seizure susceptibility. Solid arrows indicate evidence-supported relationships, whereas dashed arrows represent hypothesis-driven interactions requiring further validation. This model illustrates a conceptual framework rather than an established causal pathway.

## 5 Microbiota-targeted interventions

The framework outlined above has important translational implications. If aging increases the susceptibility of seizure-related networks to microbiota-associated inflammatory and metabolic disturbances, interventions targeting the microbiota–gut–brain axis may provide opportunities for biomarker-guided stratification and adjunctive therapeutic exploration. However, these approaches should not be considered replacements for standard antiseizure therapy, particularly because direct evidence in late-life epilepsy remains limited.

Current microbiota-targeted strategies can be broadly categorized into dietary interventions, microbial modulation, and metabolite-based approaches. Table 3 summarizes the proposed mechanisms, current evidence levels, advantages, limitations, and potential relevance of these interventions to late-life epilepsy.

**Table 3:** Summary of microbiota-targeted interventions and their potential relevance to late-life epilepsy.

Intervention	Proposed mechanism	Current evidence	Advantages	Limitations	Potential relevance to late-life epilepsy
Ketogenic diet	Modulates microbiota, SCFA-related pathways, and inflammatory signaling	Clinical epilepsy + preclinical studies	Established dietary therapy	Adherence; nutritional concerns in older adults	Potential adjunctive strategy; age-specific evidence limited
Probiotics/Prebiotics	Modulate microbial metabolism, barrier integrity, and inflammation	Mainly preclinical; limited clinical studies	Generally well tolerated	Strain- and host-dependent effects; no standardized protocols	Requires validation in late-life epilepsy
FMT	Restores gut microbial ecosystem	Proof-of-concept experimental evidence; limited clinical evidence	Broad microbiome restoration	Safety, donor selection, reproducibility	Currently investigational
Metabolite-based strategies	Restore SCFA-related signaling and barrier function	Predominantly experimental	Mechanistically targeted	Limited human evidence	Promising adjunctive approach; age-stratified validation needed

Current evidence refers primarily to microbiota-related evidence in epilepsy and should not be interpreted as established therapeutic efficacy in late-life epilepsy.

### 5.1 Ketogenic diet

The ketogenic diet is currently the best established metabolic intervention in epilepsy and may represent one of the most clinically developed approaches involving microbiota-related mechanisms. Human and animal studies suggest that ketogenic interventions have been reported to alter microbial composition and metabolite signaling in parallel with seizure improvement, potentially through combined effects on ketone metabolism, inflammatory signaling, and microbiota-related pathways (Ferraris et al., 2021; Lum et al., 2023; Meeusen et al., 2025; Özcan et al., 2025; Barros et al., 2026; Xie et al., 2026). These effects likely involve interactions among ketone signaling, microbiota remodeling, SCFA-related pathways, and inflammatory or neurotransmitter regulation (Ferraris et al., 2021; Kowalcze et al., 2025; Tang et al., 2025; Figueroa et al., 2026).

In older adults, ketogenic interventions may have relevance beyond seizure control because they may partially counteract aging-related dysbiosis, reduced barrier resilience, and chronic inflammation. Nevertheless, translation to late-life epilepsy is complicated by frailty, multimorbidity, polypharmacy, reduced physiological reserve, nutritional risk, and limited long-term tolerability, including concerns related to sarcopenia, constipation, and hepatic or renal impairment. Moreover, most direct evidence on ketogenic diet–microbiota interactions still derives from pediatric cohorts, general adult populations, or animal models rather than dedicated studies of late-life epilepsy. Accordingly, ketogenic interventions are better viewed as biologically informed adjunctive approaches rather than therapies acting solely through ketone-body elevation (Barros et al., 2026; Figueroa et al., 2026).

### 5.2 Probiotics and prebiotics

Compared with ketogenic interventions, probiotics and prebiotics provide lower-intensity and more direct approaches for microbiota modulation. Their potential value lies in modulating microbial metabolism, inflammatory signaling, and barrier-related dysfunction. Early clinical studies and meta-analytic evidence suggest that probiotic-based adjunctive treatment may reduce seizure burden in some settings (El-Sharkawy et al., 2024; Shariatmadari et al., 2024; Tan et al., 2025; Rashdan et al., 2026). Preclinical studies further support possible effects on GABA-related signaling, oxidative stress, inflammatory tone, and neurotrophic pathways (Aygun et al., 2022; Ciltas et al., 2023; Ishii et al., 2024; Wlaź et al., 2024; Braga et al., 2025).

However, reported effects remain heterogeneous and appear highly dependent on strain selection, host condition, and experimental context. Any antiseizure effects are therefore hypothesized to reflect broader modulation of microbial metabolism, inflammatory signaling, and barrier-related pathways rather than direct neural effects of individual strains.

In the aging context, probiotics and prebiotics may be better tolerated than intensive dietary interventions and may align more directly with age-related loss of microbial resilience and protective metabolite production. Nevertheless, translation to late-life epilepsy remains limited by strain specificity, impaired microbiota recovery capacity, dietary variability, constipation, low physical activity, and polypharmacy in older adults (Wang et al., 2022; Tan et al., 2025). Dedicated age-stratified studies remain lacking, and these approaches should therefore be regarded as adjunctive strategies requiring prospective validation in older populations.

### 5.3 Fecal microbiota transplantation and metabolite-based strategies

FMT and metabolite-based interventions represent more mechanistically oriented approaches. Their main value lies in determining whether epilepsy-associated dysbiosis or protective metabolic deficits can be biologically modified. FMT attempts to reconstruct the gut ecosystem at the community level, whereas metabolite-based approaches focus more directly on restoring protective signaling pathways, particularly SCFA-related mechanisms.

At present, the significance of FMT in epilepsy remains largely proof of concept. Animal studies indicate that epilepsy-associated microbial features and related phenotypes are at least partly transferable, supporting a functional role of dysbiosis in seizure-related pathology (Citraro et al., 2021). However, direct clinical evidence remains extremely limited, and current findings should primarily be interpreted as evidence for microbiota transferability rather than support for routine therapeutic application. Robust negative clinical trials of FMT in epilepsy are also lacking, indicating that the current evidence base is insufficient rather than clearly positive or negative.

Compared with ecosystem-level reconstruction, metabolite-based approaches may be more compatible with translational development. SCFA-related pathways, particularly butyrate-associated mechanisms, represent one of the better supported links among microbial remodeling, barrier dysfunction, and neuroinflammation (Ma et al., 2024a; Majumdar et al., 2024; Zhai et al., 2024; Li et al., 2026). By contrast, bile acid signaling is currently better viewed as a secondary and less epilepsy-specific metabolic interface rather than a central mechanistic axis (Ma et al., 2024a).

In older adults, FMT raises additional concerns regarding donor selection, multimorbidity, immune vulnerability, and reproducibility. By contrast, metabolite-oriented approaches, particularly those targeting butyrate-related pathways and barrier support, may be easier to standardize and integrate into biomarker-based stratification strategies. At present, these interventions are best regarded as exploratory adjunctive approaches rather than established therapies for late-life epilepsy. Their clinical relevance will require prospective age-stratified validation (Boehme et al., 2021; Parker et al., 2022; Ma et al., 2024a).

## 6 Conclusion

The evidence reviewed here supports a framework in which aging-related alterations in microbial resilience, barrier homeostasis, and neuroimmune responsiveness may increase the vulnerability of seizure-related neural networks to microbiota-associated inflammatory and metabolic disturbances. Within this perspective, microbiota-related abnormalities in older adults may become more biologically relevant not simply because dysbiosis is present, but because these disturbances occur in a host background characterized by chronic low-grade inflammation, impaired recovery capacity, and heightened sensitivity to inflammatory and metabolic stress.

Rather than representing a single causal pathway, the microbiota–gut–brain axis is more likely to function as a context-dependent modifier of seizure-related network vulnerability in late-life epilepsy. This perspective may be particularly relevant to epilepsy because seizure generation is highly sensitive to relatively small disturbances in excitatory–inhibitory balance, inflammatory signaling, barrier integrity, and glial regulation. Aging-related neuroimmune priming and reduced barrier resilience may therefore lower the stability threshold of neural networks already susceptible to hypersynchronous activity.

At the same time, direct evidence specifically addressing the microbiota–gut–brain axis in late-life epilepsy remains limited. Much of the current framework is inferred from the integration of aging biology, epilepsy research, and mechanistic microbiome studies rather than from dedicated cohorts of older adults with epilepsy. Accordingly, microbiota-targeted approaches, including ketogenic interventions, probiotics and prebiotics, fecal microbiota transplantation, and metabolite-based strategies, should currently be regarded as adjunctive and hypothesis-informed approaches rather than established therapies.

Future work will require age-stratified longitudinal cohorts that account for epilepsy etiology, integrating microbiome profiling, barrier-associated markers, inflammatory profiling, and longitudinal seizure outcomes. Within this framework, particular attention should be directed toward biomarkers reflecting key components of the microbiota–gut–brain axis, including SCFAs, LBP, inflammatory cytokines, markers of blood–brain barrier injury, and microbiome signatures. Such integrated approaches may facilitate biological stratification, improve mechanistic understanding, and help determine whether microbiota–gut–brain axis alterations contribute to clinically meaningful heterogeneity in seizure susceptibility or treatment response in older adults with epilepsy.

### 6.1 Limitations

Several limitations should be considered when interpreting the present framework. Direct evidence specifically addressing the microbiota–gut–brain axis in late-life epilepsy remains limited, and much of the current model is inferred from the integration of aging biology, general epilepsy cohorts, pediatric studies, and preclinical research rather than from dedicated cohorts of older adults with epilepsy. In addition, most available human studies remain cross-sectional and methodologically heterogeneous, limiting causal inference and making it difficult to disentangle epilepsy-related signals from the effects of diet, antiseizure medications, constipation, frailty, physical inactivity, and other age-associated confounders.

Another important limitation is the marked heterogeneity of late-life epilepsy itself. Microbiota-related inflammatory mechanisms are unlikely to contribute uniformly across cerebrovascular, neurodegenerative, tumor-related, and metabolic etiologies, and the relative importance of these pathways may differ substantially according to disease background, medication exposure, and systemic inflammatory status. Furthermore, although animal and *in vitro* studies provide important mechanistic insights, their translational relevance to older adults with epilepsy remains uncertain.

Accordingly, the present review should be interpreted as a hypothesis-generating and mechanistically integrative framework rather than as a definitive age-specific causal model. Future progress will require age-stratified and etiology-aware studies capable of integrating microbiome profiling with barrier-associated, inflammatory, metabolic, and longitudinal clinical data.
