# Correction: Aging, the microbiota–gut–brain axis, and late-life epilepsy: a hypothesis-driven review

**DOI:** 10.3389/fnmol.2026.2010236

**Published:** 2026-10-23

**Authors:** Yingsi Cao, Huiying Li, Minzheng Xu, Yuting Teng, Hai-Ying Gu

**Affiliations:** School of Health Medicine, Nantong Institute of Technology, Nantong, Jiangsu, China

**Keywords:** aging, gut dysbiosis, late-life epilepsy, microbiota–gut–brain axis, neuroinflammation

In the published article, the author Hai-Ying Gu was erroneously omitted. The correct author list reads: Yingsi Cao, Huiying Li, Minzheng Xu, Yuting Teng, Hai-Ying Gu. The citation and copyright sections have also been updated accordingly. Similarly, the **Author contributions** statement has been corrected to:

“YC: Conceptualization, Investigation, Writing – original draft. HL: Conceptualization, Supervision, Writing – review & editing. MX: Conceptualization, Investigation, Methodology, Writing – original draft. YT: Conceptualization, Investigation, Methodology, Writing – original draft. H-YG: Supervision, Writing – review & editing.”

In the published article, in the **Funding** statement, the funder “NIT Science and Technology Innovation Fund (No. KCTD012)” for this work was erroneously omitted. The **Funding** statement has been corrected to:

“The author(s) declared that financial support was received for this work and/or its publication. This research was supported by the “Health and Elderly Care” Talent-Enterprise Order Class Project of Nantong Institute of Technology (No. 6) and NIT Science and Technology Innovation Fund (No. KCTD012).”

The original version of this article has been updated.

